# *N*-(4-Nitro­pheneth­yl)formamide

**DOI:** 10.1107/S1600536810022737

**Published:** 2010-06-23

**Authors:** Li-Ping He, Xiao-Ming Yang, Dao-Lin Pang, Hui-Zhang Li, Fang Li

**Affiliations:** a College of Chemistry, Sichuan University, Chengdu 610064, People’s Republic of China; b College of Pharmaceutical Sciences, Southwest University, Chongqing 400715, People’s Republic of China

## Abstract

The title compound, C_9_H_10_N_2_O_3_, was synthesized by direct *N*-formyl­ation of 4-nitro­phenethyl­amine hydro­chloride with formic acid and sodium formate in the absence of catalyst and solvent. In the crystal structure, mol­ecules are linked by inter­molecular N—H⋯O hydrogen-bond inter­actions into chains parallel to the *a* axis.

## Related literature

For the applications and synthesis of the title compound, see: Yu *et al.* (1995 ▶); Rahman *et al.* (2010 ▶).
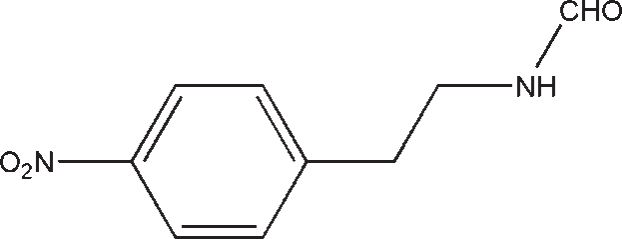


## Experimental

### 

#### Crystal data

C_9_H_10_N_2_O_3_
*M* *_r_* = 194.19
Monoclinic, 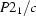

*a* = 4.4754 (1) Å
*b* = 17.6664 (5) Å
*c* = 12.1548 (4) Å
β = 93.021 (2)°
*V* = 959.67 (5) Å^3^
*Z* = 4
Mo *K*α radiation
μ = 0.10 mm^−1^
*T* = 296 K
0.42 × 0.30 × 0.28 mm

#### Data collection

Bruker SMART CCD area-detector diffractometer
Absorption correction: multi-scan (*SADABS*; Bruker, 2001 ▶) *T*_min_ = 0.681, *T*_max_ = 1.000
13857 measured reflections
2218 independent reflections
1407 reflections with *I* > 2σ(*I*)
*R*_int_ = 0.042

#### Refinement

*R*[*F* ^2^ > 2σ(*F* ^2^)] = 0.050
*wR*(*F* ^2^) = 0.157
*S* = 1.01
2218 reflections
167 parameters
All H-atom parameters refined
Δρ_max_ = 0.14 e Å^−3^
Δρ_min_ = −0.18 e Å^−3^

### 

Data collection: *SMART* (Bruker, 2001 ▶); cell refinement: *SAINT* (Bruker, 2001 ▶); data reduction: *SAINT*; program(s) used to solve structure: *SHELXS97* (Sheldrick, 2008 ▶); program(s) used to refine structure: *SHELXL97* (Sheldrick, 2008 ▶); molecular graphics: *SHELXTL* (Sheldrick, 2008 ▶); software used to prepare material for publication: *SHELXTL*.

## Supplementary Material

Crystal structure: contains datablocks global, I. DOI: 10.1107/S1600536810022737/rz2462sup1.cif

Structure factors: contains datablocks I. DOI: 10.1107/S1600536810022737/rz2462Isup2.hkl

Additional supplementary materials:  crystallographic information; 3D view; checkCIF report

## Figures and Tables

**Table 1:** Hydrogen-bond geometry (Å, °)

*D*—H⋯*A*	*D*—H	H⋯*A*	*D*⋯*A*	*D*—H⋯*A*
N2—H2*B*⋯O3^i^	0.762 (18)	2.194 (18)	2.8692 (15)	148.1 (16)

Symmetry code: (i) 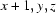.

**Fig. 1.**
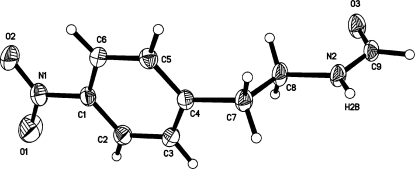
The molecular structure of the title compound, with displacement ellipsoids drawn at the 30% probability level.

## supplementary crystallographic information

### Comment

*N*-(4-Nitrophenethyl)formamide is used as an intermediate material in the synthesis of artificial chlordimeform antigen, which is applied in the immunity analysis of chlordimeform (Yu *et al.*, 1995). We report herein the crystal structure of the title compound.

In the molecule of the title compound (Fig. 1), the ethylformamide group is approximately planar (maximum deviation 0.089 (2) Å for atom C8) and perpendicular to the benzene ring (dihedral angle 89.99 (7) °). The nitro group is substantially coplanar with the benzene ring, forming a dihedral angle of 5.64 (9)°. In the crystal packing, intermolecular O—H···O hydrogen bonds (Table 1) link the molecules into chains parallel to the *a* axis.

### Experimental

A mixture of 4-nitrophenethylamine hydrochloride (4.08 g, 0.02mo1), sodium formate (2.08 g, 0.02mo1) and 88% formic acid (20 ml) was heated to reflux and the reaction was monitored by TLC (Rahman *et al.*, 2010.). 50 ml toluene was added and the solution was evaporated under reduced pressure. Dichloromethane was added to dissolve the dry residue and the extract was filtered to remove sodium chloride. The resulting solution was washed with hydrochloric acid followed by water and dried over anhydrous sodium sulfate. The solvent was evaporated under reduced pressure to get the crude product. The crude product was recrystallized with dichloromethane, and a light yellow crystalline powder was obtained. Colourless crystals suitable for X-ray analysis were obtained by slow evaporation of an ethyl acetate solution at room temperature.

### Refinement

All H atoms were located in a difference Fourier map and refined isotropically.

### Crystal data

**Table:**

C_9_H_10_N_2_O_3_	*F*(000) = 408
*M**_r_* = 194.19	*D*_x_ = 1.344 Mg m^−^^3^
Monoclinic, *P*2_1_/*c*	Mo *K*α radiation, λ = 0.71073 Å
Hall symbol: -P 2ybc	Cell parameters from 3623 reflections
*a* = 4.4754 (1) Å	θ = 2.9–26.8°
*b* = 17.6664 (5) Å	µ = 0.10 mm^−^^1^
*c* = 12.1548 (4) Å	*T* = 296 K
β = 93.021 (2)°	Block, colourless
*V* = 959.67 (5) Å^3^	0.42 × 0.30 × 0.28 mm
*Z* = 4	

### Data collection

**Table:**

Bruker SMART CCD area-detector diffractometer	2218 independent reflections
Radiation source: fine-focus sealed tube	1407 reflections with *I* > 2σ(*I*)
graphite	*R*_int_ = 0.042
phi and ω scans	θ_max_ = 27.7°, θ_min_ = 2.0°
Absorption correction: multi-scan (*SADABS*; Bruker, 2001)	*h* = −5→5
*T*_min_ = 0.681, *T*_max_ = 1.000	*k* = −23→23
13857 measured reflections	*l* = −14→15

### Refinement

**Table:**

Refinement on *F*^2^	Primary atom site location: structure-invariant direct methods
Least-squares matrix: full	Secondary atom site location: difference Fourier map
*R*[*F*^2^ > 2σ(*F*^2^)] = 0.050	Hydrogen site location: inferred from neighbouring sites
*wR*(*F*^2^) = 0.157	All H-atom parameters refined
*S* = 1.01	*w* = 1/[σ^2^(*F*_o_^2^) + (0.0811*P*)^2^ + 0.120*P*] where *P* = (*F*_o_^2^ + 2*F*_c_^2^)/3
2218 reflections	(Δ/σ)_max_ = 0.001
167 parameters	Δρ_max_ = 0.14 e Å^−^^3^
0 restraints	Δρ_min_ = −0.18 e Å^−^^3^

### Special details

**Table:**

Geometry. All e.s.d.'s (except the e.s.d. in the dihedral angle between two l.s. planes) are estimated using the full covariance matrix. The cell e.s.d.'s are taken into account individually in the estimation of e.s.d.'s in distances, angles and torsion angles; correlations between e.s.d.'s in cell parameters are only used when they are defined by crystal symmetry. An approximate (isotropic) treatment of cell e.s.d.'s is used for estimating e.s.d.'s involving l.s. planes.
Refinement. Refinement of *F*^2^ against ALL reflections. The weighted *R*-factor *wR* and goodness of fit *S* are based on *F*^2^, conventional *R*-factors *R* are based on *F*, with *F* set to zero for negative *F*^2^. The threshold expression of *F*^2^ > 2σ(*F*^2^) is used only for calculating *R*-factors(gt) *etc*. and is not relevant to the choice of reflections for refinement. *R*-factors based on *F*^2^ are statistically about twice as large as those based on *F*, and *R*- factors based on ALL data will be even larger.

### Fractional atomic coordinates and isotropic or equivalent isotropic displacement parameters (Å^2^)

**Table:**

	*x*	*y*	*z*	*U*_iso_*/*U*_eq_	
O1	0.5315 (4)	0.72237 (8)	0.42543 (13)	0.1333 (6)	
O2	0.4550 (3)	0.60396 (8)	0.40908 (10)	0.1023 (5)	
O3	0.9148 (2)	0.59710 (8)	1.14237 (8)	0.0752 (4)	
N1	0.5726 (4)	0.65789 (9)	0.45577 (11)	0.0761 (4)	
N2	1.3195 (3)	0.60193 (8)	1.04270 (10)	0.0584 (3)	
H2B	1.490 (4)	0.6035 (9)	1.0442 (13)	0.070 (5)*	
C1	0.7721 (3)	0.64453 (9)	0.55324 (11)	0.0543 (4)	
C2	0.8835 (4)	0.70538 (9)	0.61230 (13)	0.0663 (4)	
H2A	0.822 (4)	0.7538 (11)	0.5958 (13)	0.081 (5)*	
C3	1.0705 (4)	0.69170 (9)	0.70433 (12)	0.0630 (4)	
H3A	1.147 (4)	0.7334 (10)	0.7418 (12)	0.073 (5)*	
C4	1.1450 (3)	0.61872 (8)	0.73625 (10)	0.0506 (4)	
C5	1.0275 (3)	0.55894 (8)	0.67502 (11)	0.0548 (4)	
H5A	1.082 (3)	0.5077 (9)	0.6967 (12)	0.071 (5)*	
C6	0.8387 (3)	0.57117 (9)	0.58309 (11)	0.0564 (4)	
H6A	0.757 (3)	0.5311 (9)	0.5390 (13)	0.067 (4)*	
C7	1.3374 (3)	0.60491 (10)	0.84010 (12)	0.0580 (4)	
H7B	1.495 (4)	0.6414 (9)	0.8473 (12)	0.072 (5)*	
H7A	1.423 (4)	0.5531 (10)	0.8403 (13)	0.076 (5)*	
C8	1.1484 (3)	0.61015 (11)	0.93831 (12)	0.0632 (5)	
H8B	1.046 (4)	0.6620 (10)	0.9389 (14)	0.088 (5)*	
H8A	0.986 (4)	0.5736 (10)	0.9354 (14)	0.080 (5)*	
C9	1.1853 (3)	0.59714 (9)	1.13538 (12)	0.0564 (4)	
H9A	1.322 (3)	0.5926 (8)	1.1993 (13)	0.058 (4)*	

### Atomic displacement parameters (Å^2^)

**Table:**

	*U*^11^	*U*^22^	*U*^33^	*U*^12^	*U*^13^	*U*^23^
O1	0.1864 (15)	0.0955 (10)	0.1094 (10)	0.0058 (11)	−0.0736 (10)	0.0306 (8)
O2	0.1260 (11)	0.1099 (10)	0.0660 (7)	−0.0225 (8)	−0.0411 (7)	0.0019 (7)
O3	0.0479 (6)	0.1188 (9)	0.0581 (6)	0.0041 (6)	−0.0059 (5)	0.0155 (6)
N1	0.0876 (10)	0.0875 (10)	0.0510 (7)	−0.0011 (9)	−0.0159 (7)	0.0113 (7)
N2	0.0391 (6)	0.0883 (9)	0.0464 (6)	0.0004 (6)	−0.0107 (5)	0.0035 (6)
C1	0.0570 (8)	0.0655 (9)	0.0397 (6)	−0.0022 (7)	−0.0033 (6)	0.0050 (6)
C2	0.0838 (11)	0.0552 (9)	0.0585 (9)	−0.0023 (8)	−0.0116 (8)	0.0093 (7)
C3	0.0735 (10)	0.0592 (9)	0.0548 (8)	−0.0111 (8)	−0.0107 (7)	−0.0028 (7)
C4	0.0462 (7)	0.0644 (9)	0.0412 (6)	0.0000 (6)	0.0013 (5)	0.0016 (6)
C5	0.0587 (8)	0.0552 (8)	0.0502 (7)	0.0058 (7)	0.0001 (6)	0.0028 (6)
C6	0.0620 (9)	0.0601 (9)	0.0463 (7)	−0.0029 (7)	−0.0025 (6)	−0.0040 (6)
C7	0.0467 (8)	0.0772 (10)	0.0490 (8)	0.0034 (8)	−0.0064 (6)	0.0016 (7)
C8	0.0449 (8)	0.0979 (12)	0.0456 (7)	0.0021 (8)	−0.0109 (6)	0.0008 (8)
C9	0.0497 (8)	0.0704 (9)	0.0474 (7)	0.0032 (7)	−0.0149 (6)	0.0069 (7)

### Geometric parameters (Å, °)

**Table:**

O1—N1	1.208 (2)	C3—H3A	0.922 (17)
O2—N1	1.2147 (19)	C4—C5	1.380 (2)
O3—C9	1.2182 (18)	C4—C7	1.5099 (19)
N1—C1	1.4647 (18)	C5—C6	1.382 (2)
N2—C9	1.3070 (19)	C5—H5A	0.971 (16)
N2—C8	1.4542 (18)	C6—H6A	0.950 (16)
N2—H2B	0.762 (18)	C7—C8	1.502 (2)
C1—C2	1.372 (2)	C7—H7B	0.957 (17)
C1—C6	1.374 (2)	C7—H7A	0.993 (17)
C2—C3	1.382 (2)	C8—H8B	1.025 (18)
C2—H2A	0.917 (18)	C8—H8A	0.971 (18)
C3—C4	1.382 (2)	C9—H9A	0.967 (15)
			
O1—N1—O2	122.79 (15)	C6—C5—H5A	120.0 (9)
O1—N1—C1	118.37 (15)	C1—C6—C5	118.40 (14)
O2—N1—C1	118.83 (14)	C1—C6—H6A	118.9 (9)
C9—N2—C8	120.92 (13)	C5—C6—H6A	122.7 (9)
C9—N2—H2B	119.1 (13)	C8—C7—C4	109.53 (12)
C8—N2—H2B	119.8 (13)	C8—C7—H7B	109.3 (10)
C2—C1—C6	122.21 (13)	C4—C7—H7B	110.7 (10)
C2—C1—N1	119.10 (14)	C8—C7—H7A	106.8 (9)
C6—C1—N1	118.69 (13)	C4—C7—H7A	110.7 (9)
C1—C2—C3	118.31 (14)	H7B—C7—H7A	109.7 (14)
C1—C2—H2A	121.4 (11)	N2—C8—C7	113.25 (12)
C3—C2—H2A	120.0 (10)	N2—C8—H8B	107.4 (10)
C4—C3—C2	121.13 (14)	C7—C8—H8B	109.4 (10)
C4—C3—H3A	121.9 (10)	N2—C8—H8A	108.9 (10)
C2—C3—H3A	116.9 (10)	C7—C8—H8A	112.4 (10)
C5—C4—C3	118.88 (13)	H8B—C8—H8A	105.1 (15)
C5—C4—C7	120.77 (13)	O3—C9—N2	124.31 (13)
C3—C4—C7	120.26 (13)	O3—C9—H9A	122.2 (9)
C4—C5—C6	121.06 (14)	N2—C9—H9A	113.5 (9)
C4—C5—H5A	118.9 (9)		
			
O1—N1—C1—C2	5.8 (2)	C7—C4—C5—C6	176.62 (13)
O2—N1—C1—C2	−173.98 (16)	C2—C1—C6—C5	−0.8 (2)
O1—N1—C1—C6	−174.89 (17)	N1—C1—C6—C5	179.90 (13)
O2—N1—C1—C6	5.3 (2)	C4—C5—C6—C1	0.5 (2)
C6—C1—C2—C3	0.5 (3)	C5—C4—C7—C8	−96.40 (17)
N1—C1—C2—C3	179.72 (14)	C3—C4—C7—C8	80.06 (18)
C1—C2—C3—C4	0.2 (3)	C9—N2—C8—C7	−172.75 (15)
C2—C3—C4—C5	−0.5 (2)	C4—C7—C8—N2	−176.62 (13)
C2—C3—C4—C7	−177.03 (15)	C8—N2—C9—O3	2.0 (2)
C3—C4—C5—C6	0.1 (2)		

### Hydrogen-bond geometry (Å, °)

**Table:**

*D*—H···*A*	*D*—H	H···*A*	*D*···*A*	*D*—H···*A*
N2—H2B···O3^i^	0.762 (18)	2.194 (18)	2.8692 (15)	148.1 (16)

Symmetry codes: (i) *x*+1, *y*, *z*.
